# Phylogenetic analysis of avian infectious bronchitis virus S1 glycoprotein regions reveals emergence of a new genotype in Moroccan broiler chicken flocks

**DOI:** 10.1186/s12985-015-0347-8

**Published:** 2015-08-04

**Authors:** Siham Fellahi, Mehdi EL Harrak, Mariette Ducatez, Chafiqa Loutfi, Saad Ibn Souda Koraichi, Jens H. Kuhn, Slimane Khayi, Mohammed EL Houadfi, My Mustapha Ennaji

**Affiliations:** Unité de Pathologie Aviaire, Département de Pathologie et Santé Publique Vétérinaire, Institut Agronomique et Vétérinaire Hassan II, Rabat, 10000 Morocco; Société de Produits Biologiques et Pharmaceutiques Vétérinaires, Rabat, 10000 Morocco; Institut National de la Recherche Agronomique, Unité Mixte de Recherche 1225 Interaction hôtes-agents pathogènes, F-31076 Toulouse, France; Université de Toulouse, Institut National Polytechnique, Ecole Nationale Vétérinaire de Toulouse, Unité Mixte de Recherche 1225 Interaction hôtes-agents pathogènes, F-31076 Toulouse, France; Laboratoire de Biotechnologie Microbienne, Université Sidi Mohamed Ben Abdellah, Fez, 30000 Morocco; Integrated Research Facility at Fort Detrick, National Institute of Allergy and Infectious Diseases, National Institutes of Health, B-8200 Research Plaza, Fort Detrick, Frederick, MD 21702 USA; Genomique Cellulaire et Techniques Moléculaire Investigations, Université Moulay Ismail, Meknès, 50000 Morocco; Laboratoirede Virologie, Microbiologie et Qualité/ETB- Faculté des Sciences et Techniques, Mohammedia, Université Hassan II- Casablanca, Mohammedia, 20650 Morocco

**Keywords:** Infectious bronchitis virus, Phylogenetic analysis, New genotype, Morocco

## Abstract

**Background:**

Infectious bronchitis virus (IBV), a major pathogen of commercial poultry flocks, circulates in the form of several serotypes/genotypes. Only a few amino-acid changes in the S1 subunit of wild-type IBVS proteins may result in mutants unaffected by current vaccines.

**Methods:**

Partial S1 gene sequences of 3 IBV isolates of the Moroccan Italy 02 genotype from vaccinated and unvaccinated broiler chicken flocks, located in southern and central regions of Morocco, were amplified by RT-PCR, sequenced, and aligned for phylogenetic and amino-acid similarity analyses.

**Results:**

The three isolates were found genetically highly distant from known avian IBV based on partial sequences of their S1 genes: gammaCoV/chicken/Morocco/I01/2011(IBV/Morocco/01), gammaCoV/chicken/Morocco/I30/2010 (IBV/Morocco/30), and gammaCoV/chicken/Morocco/I38/2013 (IBV/Morocco/38), nucleotide sequence identities reached 89.5 % to 90.9 % among the three isolates. The deduced protein sequence identities ranged from 29.7 % (between IBV/Morocco/38 and Egypt SCU-14/2013-1) to 78.2 % (between IBV/Morocco/01 and Spain/05/866). Amino acid sequence comparison and phylogenetic analysis indicated the emergence of a new Moroccan genotype, clustering with regionally related isolates from Spain (Spain/05/866) and belonging to a new sub-genotype.

**Conclusion:**

Our sequencing results demonstrate a co-circulation of wild-type infectious bronchitis viruses in broiler chickens. These results justify permanent monitoring of circulating strains in order to rationally modify vaccination strategies to make them appropriate to the evolving field situation.

## Introduction

Infectious bronchitis (IB) is one of the most contagious diseases that affect poultry worldwide and is responsible for severe economic losses. Clinically, the disease causes respiratory distress, drop in egg production and quality in layers. Some strains of IBVare associated with nephritis [1–3].

Infectious bronchitis virus (IBV) is a member of the species *Avian coronavirus*, genus *Gammacoronavirus* (*Nidovirales*: *Coronaviridae*: *Coronavirinae*) [4]. IBV has a linear, single-stranded RNA genome of positive polarity of approximately 27 kb in length and produces enveloped virions. IBV particles consist of three major structural proteins: spike (S) glycoprotein, membrane (M) protein, and nucleocapsid (N) protein. The spike of IBV is formed by post-translational cleavage of S into two separate polypeptide components, S1 and S2 [5, 6]. S1 mediates virion attachment to IBV host cells and is a major target of neutralizing antibodies in chickens. Genotype evolution of IBV is associated primarily with changes in the S1amino-acidsequence [7, 8]. Hence, the evolutionary characterization of IBV is mainly based on the analysis of the variable S1 gene or the expressed S1 protein [9, 10].

Different IBV variants are distributed globally. Some of these variant are endemic only in particular regions, while others circulate worldwide [11]. More than 20 different IBV serotypes are differentiated worldwide that evolved from genomic insertions, deletions, substitutions, and/or RNA recombinations of the S1 gene [12–14]. This large diversity of serotypes is a major reason why commercial vaccines often fail or are only partially efficacious, and, therefore, new IB outbreaks continue to occur [1].

The first isolation and characterization of IBV from poultry flocks in Morocco was reported by El-Houadfi et al. in 1986 [15]. Six isolates were obtained, of which isolates designated D, E, F, H, and M were found to be serologically related to the Massachusetts (Mass) serotype, whereas the sixth isolate, G, differed from the Mass serotype and other serotypes known at the time. Importantly, El-Houadfi et al. demonstrated that Mass-based IBV vaccines provided poor protection against infection with isolate G [15]. In 2004, Alarabi conducted a study to determine the relationship between IBV and nephropathogenic disease outbreaks observed in broiler flocks in Morocco between 1996 and 2000. Three different groups of IBV isolates were identified using RT-PCR coupled with restriction fragment length polymorphism (RFLP). Group I belonged to the Mass serotype, whereas groups II and III were distinct. Isolate 12/97 of group III, found to be closely related to isolate G, caused more severe kidney lesions and higher lethality in experimentally infected animals compared to isolate 7/97 of group II [16]. In 2005, El Bouqdaoui et al., while studying nephropathogenic IBV using RT-PCR and RFLP techniques, identified five genotypes, three of which differed from vaccine strains [17].

The Moroccan poultry industry has developed significantly under the framework "Plan Green Morocco" and meets the growing domestic consumer demand for poultry products, including meat and eggs The industry generates a turnover of around 30 billion dirhams/year, totaling mean investments of around 8.7 billion dirhams/year. The official data regarding the general poultry rearing systems in Morocco are: broiler production, 450 million/year (among 6,800 farms); layers: 20 million/year (250 farms); turkeys: 12 million/year (25 % imported d-old chicks); broiler breeders: 3.2 million/year (70 % imported from Spain and 30 % from other European countries); 48 hatcheries. The Interprofessional Federation of Poultry Sector (FISA) aims to develop exports of poultry products in particular to Northern and Western Africa. FISA is affiliated with Association of Moroccan Exporters (ASMEX**)** under the objective of promoting poultry product export in Africa, including chicks, hatching eggs, and compound feed for poultry. In 2012, 6.7 million hatching eggs and 1.9-million–d-old chicks were exported to Mauritania, Mali, Cameroon, Côte d'Ivoire, and Senegal, and 25,000 tons of compound feed were exported to Mauritania. Breeder chicks (182,000) were imported in January 2014 (an increase of 28 % compared to 2013). However, imports of layer and broiler breeder and turkey poults declined in recent years, mainly due the development of local production.

Three IBV vaccines are used in Morocco to protect the industry: 793B, Arkansas, and Mass. The Mass type has been used as of 1970 (H120, Ma5, and modified Mass strains), whereas 793B (4/91 and CR88 strains) and Arkansas vaccines were introduced in 2000 and 2013, respectively. New IBV serotypes and genotypes can emerge as a result of only very few changes in the amino acid sequence of the S protein. Vaccination programs largely rely on the use of the IBV Massachusetts strain, which is also the most commonly used IBV vaccine strain in Morocco. However, despite the use of this vaccine, the common presences of IB in vaccinated chickens continue to have a major negative economic impact [18]. In this study, IB was diagnosed between January 2010 and December 2013 in southern and central regions of Morocco. Suspected IBV infections were found in 47 commercial broiler chicken farms. Among these, three flocks showed severe clinical signs and mortality. The aim of the present study is to characterize a new emerging genotype of IBV from these outbreaks using molecular techniques, and to determine the relationship to reference IBV strains by nucleotide sequence analysis.

## Results

### Case history

Infectious bronchitis was diagnosed between January 2010 and December 2013 in southern and central regions of Morocco. Suspicious IBV infections (prominent respiratory disease) were found in broiler chickens from 47 commercial farms. The flocks had been vaccinated against Newcastle disease and infectious bursal disease (Table 1). Among these farms, three flocks had severe respiratory signs and experienced increased mortality. The first flock, located in southern Morocco (Marrakech), was 26 d old and was the only flock that had been vaccinated with the H120 live IB vaccine by spray on d 7 and 21. The second flock, located in central Morocco (Khamisset), was 24 d old, and the third flock, located also in central Morocco (Rabat) was 20 d old. Clinical presentation started with respiratory depression and distress, including nasal discharge, sneezing, coughing, and rales. Other signs included conjunctivitis and watery eyes. Within 10 d after the appearance of the disease, lethality increased from 10 % to 30 % of the total flocks. Post-mortem examination of dead birds revealed increased tracheal mucus, slight congestion, and presence of catarrhal exudates in the nasal turbinate and trachea.

**Table 1:** Characterization of Moroccan infectious bronchitis virus isolates from broiler chickens

Isolate designation	Age, d	Time of vaccination, d, and (virus vaccine)	Date of isolation	GenBank accession no.
Moroccan 30	26	7 and 21 (IBV)^a^	May 2010	KJ701020
		14 (NDV)^b^		
		18 (IBDV)^c^		
Moroccan 38	24	7 (NDV)^b^	December 2013	KJ701019
		22 (IBDV)^c^		
Moroccan 01	20	5 (NDV)^b^	April 2011	KM594187
		18 (IBDV)^c^		

^a^IBV = Infectious bronchitis virus, ^b^NDV = Newcastle disease virus, ^c^IBDV = Infectious bursal disease virus

### IBV isolation and identification

We obtained a total of 47 Moroccan field isolates of IBV from the 47 affected farms and were able to distinguish them based on sequences of the hypervariable region (HVR) 3 of their S1 regions (nucleotides 700–1095). Of those isolates, three isolates, classified as the Moroccan Italy 02 genotype, underwent further sequencing to include all three hypervariable S1regions (nucleotides 1–1,095). These three IBV strains were isolated from trachea, lungs, and kidneys of broiler chickens. Between the first and fourth passage, virus propagation from the three suspect samples in specific pathogen-free eggs resulted in characteristic IBV lesions (e.g., curled and dwarfed embryos with head pressed over the feet and covered by a thickened amnion)in 90 %–100 % of the embryos. Results of both RT-PCR targeting the IBV N gene and RT-PCR using primers amplifying a partial sequence of the IBV S1 gene were positive, confirming the identity of the three isolates. The amplified partial S1 fragments (approximately 700 bp in length) of the three isolates were sequenced. The sequences were submitted to GenBank and assigned the accession numbers KJ701019, KJ701020 and KM594187 for isolates gammaCoV/chicken/IBV/Morocco/I38/2013 (IBV/Morocco/38), gammaCoV/chicken/IBV/Morocco/I30/2013 (IBV/Morocco/30), and gammaCoV/chicken/IBV/Morocco/I01/2013 (IBV/Morocco/01), respectively (Table 1).

### Sequence analysis

Partial S1 gene sequences of the three IBV isolates were sequenced. The nucleotide sequence and deduced amino acid sequences (Table 2) of these IBV isolates were blasted and compared with the reference and variant strain sequences retrieved from GenBank from different regions of the world. IBV isolates (IBV/Morocco/30, IBV/Morocco/38, and IBV/Morocco/01) were found to have different and unique partial S1 sequences compared to reference strains. The sequences of the three isolates have a nucleotide sequence identity between 91.6 % (IBV/Morocco/38 and IBV/Morocco/01) and 94.8 % (IBV/Morocco/30 and IBV/Morocco/01) when compared to each other and from 29.7 % (IBV/Morocco/38 or IBV/Morocco/30 and Egypt SCU-14/2013-1)to 78.2 % (IBV/Morocco/01 and Spain/05/866) when compared to non-Moroccan IBV strains.

**Table 2:** Nucleotide and amino acid identities of IBV Moroccan isolates and reference strains

	S1 gene nucleotide identity (%)																		
Strains	1	2	3	4	5	6	7	8	9	10	11	12	13	14	15	16	17	18	19
1. IBV/Morocco/38	100	93.5	91.6	74.3	74	73.8	73.2	72.6	65.6	61.8	62	56.1	61.5	53	46.3	62	31.5	29.7	31.5
2. IBV/Morocco/30	90.9	100	94.8	77.7	77.7	77.4	76.7	76.2	68.7	65.2	65.4	58.5	65.1	49.5	48.2	65.4	31.46	29.7	33
3. IBV/Morocco/01	89.4	90.9	100	78.2	77.8	77.8	76.8	76.3	69.9	65	65	59.1	65.7	48.8	49.3	65	33.7	31.8	33.7
4. Spain/05/866	73.6	75.8	75.6	100	98.3	98.3	93.7	92.9	77.5	71.3	71.6	63.9	71.6	41.6	52.9	71.6	75.2	35.5	37
5. Spain/04/22-1	73.2	76.8	74.6	96.5	100	97.7	93.9	93.5	78.1	70.8	71	63.7	71.4	41.8	52.7	71	75.2	35.5	37
6. Spain/05/221	72.7	76.3	75.1	95.4	94.8	100	93.1	93.1	78.2	71.4	71.6	64.3	71.6	42.1	53.1	71.5	75	35.7	37
7. Italy-02	71.3	74.8	73.6	89.7	91.4	89.7	100	97.1	74.6	71	71.2	65.1	69.8	4.3	52.3	71.2	73.3	35.3	36.4
8. It/497/02-1	69.4	73.3	71.6	90.2	92	91.4	93.1	100	75.6	70.4	70.6	64.2	70.4	41.8	52.1	70.6	74.1	34.9	35.7
9. 4/91	63.6	65.3	66.5	76.1	76.1	76.7	72.2	72.2	100	74.14	74.3	60.6	73.4	38.4	48.9	74.3	71.5	34.9	35.9
10. H120	58.8	61.8	61.4	68.4	67.2	66.1	66.1	64.9	65.3	100	99.4	61.1	72.2	35.9	45.9	99.4	69.2	33.1	35
11. Ma5	59.3	62.3	61.9	68.9	67.8	66.7	66.7	65.5	65.3	99.4	100	61.3	72.4	36.1	46.1	100	69.3	33.1	35
12. D274	55.1	57.3	58.4	66.5	65.9	67	67.6	65.9	61.9	58.9	58.9	100	58.8	33.8	42.4	61.3	61.1	27.9	29.5
13. Qx	60	62	62.6	68.9	67.8	66.7	67.8	66.1	69.3	72	72.6	60.2	100	37.3	47.9	72.4	70.2	32.1	32.8
14. TN556/07	52.7	48.5	48.1	42.1	42.5	42.9	40.8	42.1	41	34.4	34.9	36.1	37.4	100	58.7	36.1	38.7	22	22.2
15. TN296/07	37.3	38.6	39.5	45.9	44.8	44.8	44.5	44.3	43.7	38.5	39	38.2	40.7	41.6	100	46.1	49.2	28.2	28.5
16. NGA/A116E/2006-1	59.3	57,9	60,41	68.9	67.8	66.7	66.7	65.5	65.3	99.4	99.4	58.9	72.6	34.9	39	100	69.3	33.1	35
17. Ark/C6d	61.3	63.8	61.9	73.7	73.7	73.2	72.1	72.1	67.1	63.9	64.4	62.5	68.9	39.4	42.4	64.4	100	34.5	36.4
18. Egypt/SCU-14/2013-1	12.9	14.5	13.6	16.9	17.5	16.9	16.4	16.4	14.3	16.5	17	12.6	17.1	10.4	11.6	17	16.1	100	81.8
19. Egypt/Beni-Suef/01	13.8	14	14.6	18	18.6	18	17.5	17.5	15.4	17	17.6	14	18.8	10.7	11.4	17.6	17.2	81.3	100
	S1 protein amino acid identity (%)																		

**Fig. 1:**
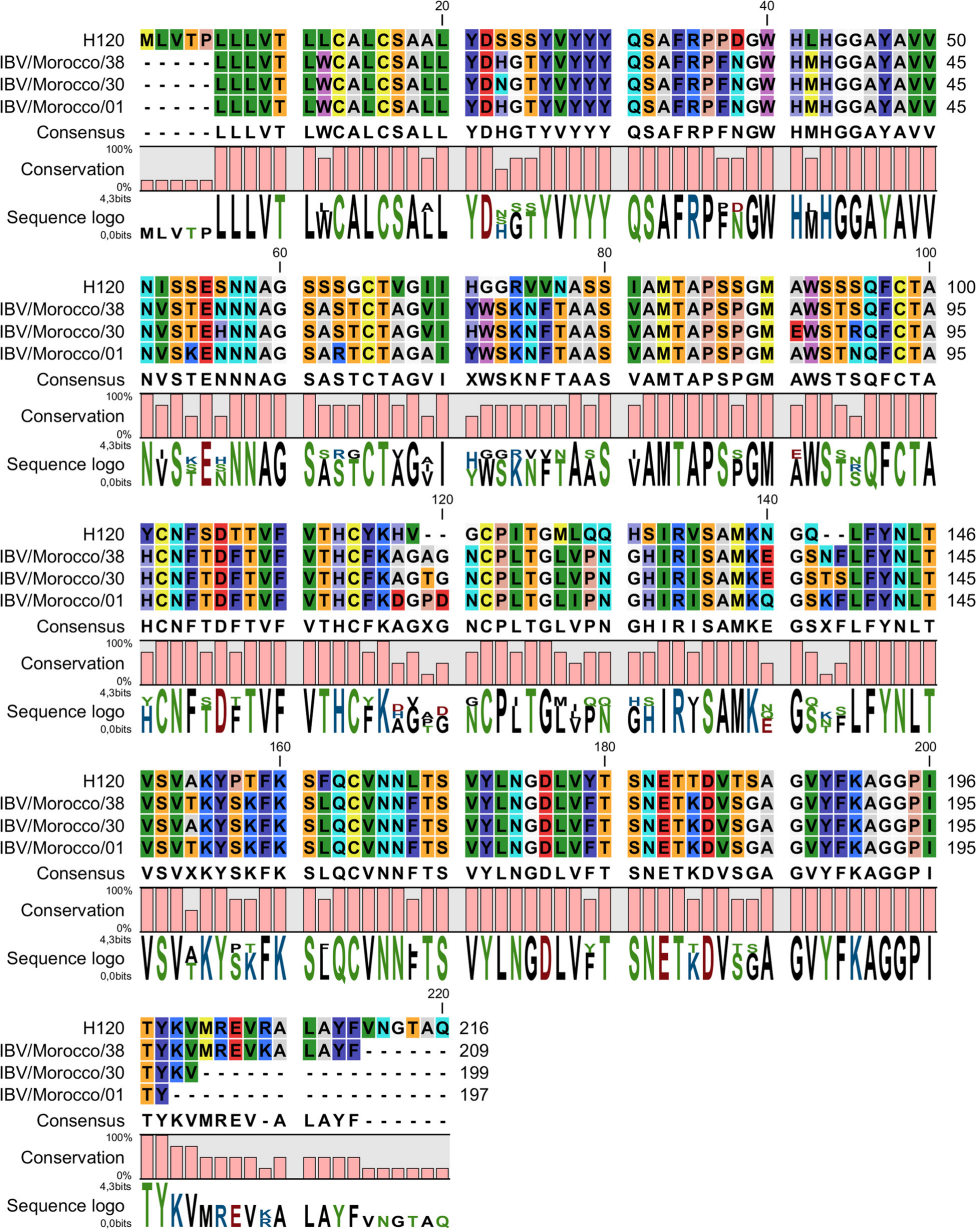
Alignment of partial amino acid S1 sequences of the obtained Moroccan IBV isolates with the H120 referencestrain (amino acids 1–216)

The deduced amino-acid sequence identities of Moroccan IBV isolates ranged from89.4 % (between IBV/Morocco38 and IBV/Morocco/01) to 90.9 % (between IBV/Morocco/38, IBV/Morocco/30and IBV/Morocco/01) when compared to each otherand from 12.9 % (between IBV/Morocco/38 and Egypt SCU-14/2013-1)to 76.8 % (between IBV/Morocco/30 and Spain/04/22-1 of the Spanish Italy 02 genotype), [19] when compared to non-Moroccan IBV strains (Table 2). The alignment of the Moroccan IBV isolates amino-acid sequences with that of reference strain H120 identified the most variable regions in residues 62–77 and 117–131 (numbering in reference to the H120 strain, Fig. 1). Insertions in the partial S1 subunit of the three Moroccan strains were found at positions119, 120, 143, and 144 (Fig. 1).

The partial S1 protein sequences of the Moroccan isolates were 69.4 % to 76.8 % homologous to those from genotype Italy-02 (Italy 02 [AJ457137]; Italy 497/02-1 [DQ901377]; Spain/05/866 [DQ386102]; Spain/04/22-1 [DQ386100]; Spain/04/221[DQ386103]) but were <66.5 % homologous to the remaining reference strains (Table 2).

**Fig. 2:**
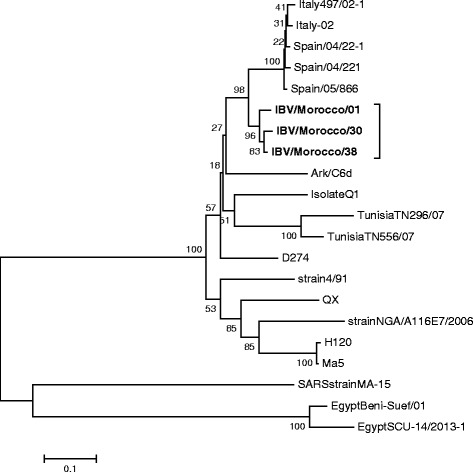
Phylogenetic relationships of the obtained Moroccan isolates and selected reference strains based on partial S1 nucleotide sequences determined using MEGA 5.0 with the Clustal W method. Numbers along the branches refer to bootstrap values

Phylogenetic trees were constructed from the nucleotide and deduced amino-acid sequences of the partial S1 glycoprotein genes of Moroccan IBV isolates and non-Moroccan IBV referenced strains. The three Moroccan IBV isolates and Spanish isolates of the Italy 02 genotype formed a common branch (Fig. 2), but they clearly clustered into 2 distinct sub-genotypes within the group. The sequence analysis of the partial S1 gene demonstrated that Moroccan isolates represent a unique sub-genotype compared to other reference strains of various countries and are distantly related to African, European, and American strains.

## Discussion

IBV was detected in poultry in Morocco in the 1980s, and in more recent years, outbreaks of IB were reported repeatedly [15–17]. The present study suggests that a new genotype, represented by the three new IBV isolates described here, has been circulating in Morocco since at least 2010 (IBV/Morocco/30, accession number: KJ701020).

Phylogenetic analyses comparing the partial S1 gene sequences of the three Moroccan field isolates with those of reference and variant strain sequences revealed that the Moroccan isolates were 69.4 % to 76.8 % homologous with Spanish isolates of the Italian 02 genotype [19]. This similarity can be explained by the geographical proximity and close transboundary of Morocco and Spain. Moreover, these two countries have an ancient history of commercial exchanges. In addition, Spain is the most frequent country that exports breeder chicks to Morocco (exports areunder the control of Office National de Sécurité Sanitaire des ProduitsAlimentaires, http://www.onssa.gov.ma/).

The deduced amino-acid sequences of Moroccan IBV isolate proteins demonstrated that the Moroccan isolates represent a unique genotype compared too ther reference strains of various countries. These Moroccan isolates are only distantly related to African, European, and American strains. The origin of this novel genotype remains to be identified. In addition to population diversity [20–22], widespread use of various vaccines made from heterotypicIBVs in the field, recombination as a consequence of mixed infections, or a decrease of dominant serotypes as a result of vaccination may play important roles in the emergence of such novel genetic variants in Morocco.

Alignment of the partial S1 glycoprotein sequence of Moroccan IBV isolates with thatof published non-Moroccan IBV strain, H120, identified most sequence variations between residues 62–77 and 117–131 (Fig. 1). Both of these sequence regions correspond to known HVR, mainly HVR1residues 50–69, and HVR2, residues 117–131, of the IBVS1 gene. HVR1 and HVR2 contain sequences that have been associated with specific IBV serotypes [7, 23] andserotype-specific neutralizing epitopes [8]. The nucleotide S1sequences of IBV serotypes commonly differ by 20 to 25 % [24], but amino acid S1 sequences of some serotypes differ by as little as 2 % [25]. The HVRs are associated with two separate viral neutralizing and conformationally-dependent epitopes [7, 26].

The insertions of four amino acids in the S1 subunit of the three Moroccan strains possibly havesome significance relatedto S1 functions (e.g., neutralization phenotypes, virus tropism) [7, 25, 27–29]. Further studies are needed to determine the role of theseinsertions in the S glycoprotein [30], which might be related to reported changes in the clinical manifestations of IB.

In the phylogenetic tree shown in Fig. 2, the Moroccan IBV isolates formed a distinct cluster withthe Spanish isolates of the Italy 02 genotype (supported by a high bootstrap value: 96). The Spanish IV genotype was previously established as a new genotype based on the mosaic nature of its S1 gene. Viral neutralization studies revealed Italy 02 and Spanish IV genotypes are subtypes within a common serotype [19].

## Conclusion

This molecular study has revealed that a new genotype can emerge as a result of only a few changes in the amino acid composition in the S1subunit of the virus Sprotein [25]. This is the first report of identification and genotyping of emergent IBV isolates in Morocco, which indicate the circulation of previously unknown IBV variants in both vaccinated and unvaccinated broiler chickens.

Our data provide evidence of a new emerging genotype in Morocco. Three IBV field isolates were different from published referenced strains in nucleotide and amino acid sequences and clustered into a separate branch based onphylogenetic analysis. All Moroccan IBV variants presented specific nucleotide and amino acid sequence insertions in the S1 gene in comparison to the Massachusetts vaccine strain that might be associated with the occurrence of clinical disease in vaccinated flocks. Further investigation related to cross protection and pathogenicity of this new genotype detected in this country are needed.

## Methods

### Tissue sampling from chickens with suspected IBV infection

Tissue samples from trachea, lungs, and kidneys were obtained from 10 birds from each flock, placed on ice, and transferred to the laboratory in sterile transport medium containing 5 % antibiotics (20,000 U/ml of penicillin, 10,000 μg/ml of streptomycin, and 5,000 μg/ml of kanamycin). These samples were used for virus isolation.

### Virus isolation

Virus isolation was performed by inoculation of 9–11-d-old, embryonated, specific pathogen-free hen’s eggs with 200 μl of 10 % tissue homogenates. Eggs were incubated at 37 °C and candled daily. Embryos that died within 24 h after inoculation were discarded. On d 2 after inoculation, three eggs were chilled at 4 °C for 2 h, and the allantoic fluids were harvested, centrifuged at 8,000 rpm (xxx x *g*) for 15 min, and stored at −80 °C. The remaining eggs were further incubated for 5 more d and observed for typical embryonic lesions of IBV infection, such as dwarfing, stunting, and curling. Stored allantoic fluids were further passaged three times in specific pathogen-free eggs; the allantoic fluids were harvested and tested for the presence of IBV using rRT-PCR.

### RNA extraction

Viral RNA was extracted from infected allantoic fluids using the MagMax Express semi-automatic magnetic particle extractor (Life Technologies, Grand Island, NY) according to the manufacturer’s instructions. Allantoic fluid samples and reagents were dispensed into X-well microplates prior to magnetic particle processing. After processing, each sample of extracted RNA recovered from the wells of the plates was subjected toRT-PCR amplification.

### Real time RT-PCR for nucleocapsid gene detection

The oligonucleotides and the probe used for initial screening of IBV were described previously [31]. These oligonucleotides and the probe target the relatively conserved IBV N gene at nucleotide positions 741–1077 of the IBV Massachusetts H120 reference strain (GenBank accession number: AM260960). RNase free water and Newcastle disease virus strain (HB1) were included as negative controls. One-step RT-PCR was performed with 12.5 μl of 2× RT-PCR buffer mix, 0.5 μlof MgSO_4_ (50 mmol/l), 0.5 μl of Rox reference dye (25 mmol/l, Life Technologies), 0.5 μl of Moloneymurine leukemia virus reverse transcriptase (200 U/μl), 0.5 μlof Taq DNA polymerase (Life Technologies), 0.5 μl of primers (0.2 μmol/l), 0.25 μl of probe (0.1 μmol/l), and 5 μl of RNA template to make a final volume of 25 μl.The reaction was carried out using a StepOne Plus real-time PCR system (Smart Cycler, Cepheid, Sunnyvale, CA) at 50 °C for 15 min, 95 °C for 5 min, and followed by 40 cycles of 9 °C for 15 s; 60 °C for 45 s; 72 °C for 30 s, and a final extension step of 74 °C for 5 min. Amplifications were recorded and analyzed, and the threshold cycle (C_t_) was determined with StepOne software (Smart Cycler).

### RT-PCR for S1 gene detection and DNA sequencing

Amplification of partialS1 gene sequences by RT-PCR was performed using the forward S1S15’ mod (5’-TGA-AAA-CTG-AAC-AAA-AGA-3’) and reverse S1CK2 (5’-CNG-TRT-TRT-AYT-GRC-A-3’) primers [32]. RT-PCR was performed using the Applied Biosystems kit protocol (Life Technologies). The RT reaction was performed in a 20 μl reaction mixture containing 2 μl of buffer (10×), 2.5 μl of MgCl_2_ (25 mmol/l), 2.5 μl of dNTP (10 mmol/l), 0.75 μl of each primer (10 μmol/l), 10.2 μl of sterilized water, 0.5 μl of RNAase inhibitor (20 U/μl), 0.3 μl of RT (50 U/μl), and 0.5 μl of Gold Taq polymerase (5 U/μl). The PCR products were analyzed on a 1 % agarose gel.

Partial RT-PCR products of the S1 gene (700 bp) containing HVR1, HVR2, and HVR3 were purified using the Gene Clean Kit (ExoSAP-IT, Affymetrix, Santa Clara, CA) according to the manufacturer’s instructions. Purified PCR products were used as templates for sequencing using the BigDye® Terminator v1.1 Cycle Sequencing Kit (Life Technologies). The second purification step was performed using the Big Dye XTerminator Purification Kit (Life Technologies). Purified PCR products were sequenced from both directions using the same primers (S15’ mod and CK2).

### Nucleotide sequence and deduced amino-acid-sequence analyses

Assembly and analysis of sequence data were conducted using the BioEdit Software version 5.0.9 [33]. The open-source BLAST program (National Center for Biotechnology Information, Bethesda MD, http://blast.ncbi.nlm.nih.gov/Blast.cgi) was used for sequence comparison. Nucleotide sequence and deduced amino acid sequences were aligned using ClustalW and MEGA software Version 6.0 [34]. Phylogenetic analyses and tree construction for partial S1 glycoprotein gene sequences were generated using the neighbor-joining method with 1,000 bootstrap replicates with MEGA.

### Genbank accession numbers of reference IBV variants

Ark/C6d (EU283056); D274 (X15832); Egypt/Beni-Suef/01 (JX174183); Egypt/SCU-14/2013-1 (KF731612); H120 (M21970); Italy-02 (AJ457137); Italy It/497/02-1 (DQ901377); Ma5 (AY561713); QX (AF193423); Spain/04/22-1 (DQ386100); Tunisia TN296/07 (FJ716133); Tunisia TN556/07 (FJ716132); Spain/05/866 (DQ386102); Spain/04/221 (DQ386103); Isolate Q1 (AF286302); SARS (D14056); Strain 04/91 (AF093794);NGA/A116E7/2006 (FN430415).

## Abbreviations

IBV: Infectious bronchitis virus
IBV/Morocco/01: gammaCoV/chicken/Morocco/I01/2011
IBV/Morocco/30: gammaCoV/chicken/Morocco/I30/2010
IBV/Morocco/38: gammaCoV/chicken/Morocco/I38/2013
IB: Infectious bronchitis
S: Spike
M: Membrane
N: Nucleocapsid
HVR: Hypervariable regions
